# The Gene expression Grade Index: a potential predictor of relapse for endocrine-treated breast cancer patients in the BIG 1–98 trial

**DOI:** 10.1186/1755-8794-2-40

**Published:** 2009-07-02

**Authors:** Christine Desmedt, Anita Giobbie-Hurder, Patrick Neven, Robert Paridaens, Marie-Rose Christiaens, Ann Smeets, Françoise Lallemand, Benjamin Haibe-Kains, Giuseppe Viale, Richard D Gelber, Martine Piccart, Christos Sotiriou

**Affiliations:** 1 Institut Jules Bordet, Université Libre de Bruxelles, Brussels, Belgium; 2 IBCSG Statistical Center, Dana-Farber Cancer Institute, Boston, MA, USA; 3 U.Z. Gasthuisberg, Katholieke Universtiteit Leuven, Leuven, Belgium; 4 European Institute of Oncology, Milan, Italy; 5 IBCSG Statistical Center, Dana-Farber Cancer Institute, Frontier Science and Technology Research Foundation, Harvard School of Public Health, Boston, MA, USA

## Abstract

**Background:**

We have previously shown that the Gene expression Grade Index (GGI) was able to identify two subtypes of estrogen receptor (ER)-positive tumors that were associated with statistically distinct clinical outcomes in both untreated and tamoxifen-treated patients. Here, we aim to investigate the ability of the GGI to predict relapses in postmenopausal women who were treated with tamoxifen (T) or letrozole (L) within the BIG 1–98 trial.

**Methods:**

We generated gene expression profiles (Affymetrix) and computed the GGI for a matched, case-control sample of patients enrolled in the BIG 1–98 trial from the two hospitals where frozen samples were available. All relapses (cases) were identified from patients randomized to receive monotherapy or from the switching treatment arms for whom relapse occurred before the switch. Each case was randomly matched with four controls based upon nodal status and treatment (T or L). The prognostic value of GGI was assessed as a continuous predictor and divided at the median. Predictive accuracy of GGI was estimated using time-dependent area under the curve (AUC) of the ROC curves.

**Results:**

Frozen samples were analyzable for 48 patients (10 cases and 38 controls). Seven of the 10 cases had been assigned to receive L. Cases and controls were comparable with respect to menopausal and nodal status, local and chemotherapy, and HER2 positivity. Cases were slightly older than controls and had a larger proportion of large, poorly differentiated ER+/PgR- tumors. The GGI was significantly and linearly related to risk of relapse: each 10-unit increase in GGI resulted in an increase of approximately 11% in the hazard rate (p = 0.02). Within the subgroups of patients with node-positive disease or who were treated with L, the hazard of relapse was significantly greater for patients with GGI at or above the median. AUC reached a maximum of 78% at 27 months.

**Conclusion:**

This analysis supports the GGI as a good predictor of relapse for ER-positive patients, even among patients who receive L. Validation of these results, in a larger series from BIG 1–98, is planned using the simplified GGI represented by a smaller set of genes and tested by qRT-PCR on paraffin-embedded tissues.

## Background

Most breast cancer patients whose tumors express the estrogen receptor (ER) receive endocrine therapy. Despite ER status being one of the most reliable biomarkers used today to predict response to endocrine therapy, such as tamoxifen or an aromatase inhibitor, a significant proportion of women still relapse, which indicates the need for additional predictive markers.

Several studies have reported that breast cancer is a molecularly heterogeneous disease and that distinct gene expression patterns are particularly evident in women within the subgroup of ER-positive breast cancers (reviewed in [1]). These studies have consistently shown, on the basis of hierarchical clustering of gene expression profiles, that ER-positive breast cancers can be classified into molecular subtypes (mainly luminal A and B) and that these subtypes are associated with a different clinical outcome, suggesting a molecular basis behind the clinical heterogeneity. Unfortunately, the classifications generated by this cluster analysis are at present not useful for the clinical setting, since there is currently no operational definition of what constitutes each luminal subtype.

Our group recently developed a Gene expression Grade Index (GGI) score based on 97 genes mainly involved in cell cycle regulation, proliferation and differentiation and consistently differentially expressed between low and high grade breast carcinomas [2]. Interestingly, the GGI was not only able to reclassify patients with histological grade 2 tumors into two groups with distinct clinical outcomes similar to those of histological grade 1 and 3, but also to define two molecular subgroups within ER-positive breast cancers, in a reproducible and quantitative manner, that were highly comparable to the previously described luminal A and B classification [3]. Indeed, the samples previously classified as luminal A or B were associated with significantly different GGI values across the different populations evaluated, with all of the ER-positive luminal A subtypes, which had the best clinical outcome, being associated with low GGI values and the luminal B tumors having significantly higher GGI values. We also showed that these two subtypes were associated with statistically distinct clinical outcome in both systemically untreated and tamoxifen-treated populations. Given these results, it appeared crucial to understand whether patients with a high GGI would benefit from alternative anti-estrogen agents, such as aromatase inhibitors, which have globally shown superiority over tamoxifen [4-8] or would need a completely different treatment strategy. Thus, we aimed to investigate the ability of the GGI to predict relapses in postmenopausal women with hormone receptor-positive breast cancer who were treated with tamoxifen (T) or letrozole (L) within the BIG 1–98 trial.

## Methods

### Patients

The design and conduct of the BIG 1–98 study have been described elsewhere [9]. Briefly, the BIG 1–98 trial consists of four treatment groups that compare 5 years of monotherapy with letrozole (L) or tamoxifen (T), and sequential administration of one drug for 2 years followed by the other drug for 3 years. Patients with cancer relapse from sites with available frozen material, needed for gene expression profiling, were identified from the BIG 1–98 database (version of February 2007). Only two Belgian sites had frozen material. Relapses (cases) were defined as either local, contra-lateral breast, regional, distant soft tissue, bone, or distant viscera and were identified from patients randomized to receive either monotherapy or from the switching treatment arms for whom relapse occurred before the switch. There were 14 patients with relapse from these two hospitals. Each was randomly matched with four controls based upon nodal status (negative (N-/Nx) or positive (N+)) and treatment (T or L), resulting in a listing of 70 patients. The investigators received only a list of patient identification numbers with no additional information. This study was approved by the BIG 1–98 Steering Committee and by the local ethics committee.

### Gene expression analysis

Frozen samples from the cases and controls selected by the International Breast Cancer Study Group (IBCSG) Statistical Center were collected in the two hospitals and sent to the Translational Research Unit from the Institut Jules Bordet where the samples were further processed. Isolation of RNA was performed using the Trizol method (Invitrogen) according to the manufacturer's instructions and purified using RNeasy mini-columns (Qiagen, Valencia, CA). The quality of the RNA obtained from each tumor sample was assessed based on the RNA profile generated by the bioanalyzer (Agilent Inc). RNA amplification, hybridization and image scanning were done according to standard Affymetrix protocols. We used the Affymetrix Human Genome U133-2.0 plus GeneChip, which contains almost 50,000 probe sets representing more than 47,000 transcripts, derived from approximately 39,500 well-substantiated human genes. The GGI scores were defined as in Sotiriou et al. [2] by the Institut Jules Bordet, blinded to the clinical data, and sent to the IBCSG statistical office. The raw gene expression data, together with the patient's characteristics are publicly available on GEO , with accession number GSE16391.

### Pathology

The IBCSG Central Pathology Laboratory performed central review of paraffin-embedded primary tumor specimens for HER2 by IHC and fluorescence in-situ hybridization (FISH) [10]. Tumors were considered to be HER2-positive if amplified by FISH, or in a few cases with unevaluable FISH results, if IHC = 3+.

### Statistical analyses

Cases and controls were compared descriptively based upon demographics (menopausal status, age), tumor characteristics (tumor size and grade, nodal status, ER/PgR status, HER2 positivity), therapy received (breast-conserving surgery, mastectomy, radiotherapy, adjuvant or neoadjuvant chemotherapy), length of follow-up, and GGI. Comparisons of disease and demographic characteristics at baseline were conducted using Wilcoxon rank-sum tests for continuous variables and Fisher's exact test for categorical variables. Data analysis was based on Kaplan-Meier estimation and general principles of the Cox model. To evaluate the independent prognostic value of the GGI, multivariable proportional hazards regression models were employed. Models were stratified by combinations of the matching criteria, nodal status and treatment, and results reported using hazard ratios with 95% confidence intervals. Multivariable proportional hazards regression was also used to explore the prognostic value of GGI within subgroups defined by treatment or nodal status. Performance of the GGI was summarized as a continuous predictor and divided at the median

To assess the predictive accuracy of the GGI, time-dependent sensitivity and specificity and time-dependent ROC and AUC curves were constructed using the incident/dynamic definitions of Heagerty and Zheng [11]. The areas under the time-specific ROC curves, AUC(t), were plotted as a function of time to characterize temporal changes in accuracy of the GGI marker. An R/S-plus package, risksetROC, was used to handle the computations and was available for download through Dr. Heagerty's website [12].

All the statistical analyses were performed by the IBCSG Statistical Center (AG-H and RDG).

## Results

### Description of the study population

Frozen tissue was available and analyzable for 48 patients out of the original list of 70 (see methods for selection procedure), 10 cases (relapses) and 38 controls (non-relapses). Due to the limited amount of available tissue, samples from controls that did not have results from their original matched case were paired, where applicable, with another case having the same matching characteristics. Five controls were reassigned and resulted in two cases each matched with 6 controls and one case matched with five. Controls were reassigned before any analyses or data comparisons were conducted. Table 1 summarizes the number of relapses and matching criteria for samples with GGI. Five patients with relapse (50%) had tumors in the distant viscera, two (20%) recurred in the bone, two (20%) had contra-lateral breast cancer, and one (10%) had a local recurrence. The median time to relapse among cases was 23.8 months [range: 11 to 49 months].

**Table 1:** Relapse status by nodal status and treatment for which frozen samples and GGI score were available.

	**Relapse (Cases)**	**No relapse (Controls)**	**Total**
**N-/Nx, L**	3	9	12

**N-/Nx, T**	1	5	6

**N+, L**	4	12	16

**N+, T**	2	12	14

**Total**	10	38	48

N-: node-negative; N+: node-positive; Nx: unknown nodal status. L: letrozole and T: tamoxifen

Tables 2 and 3 summarize the demographic and tumor characteristics of cases and controls. Cases and controls were comparable with respect to menopausal status, nodal status, local therapy, HER2 positivity, and adjuvant or neoadjuvant chemotherapy. Cases were slightly older than controls, with median ages of 62.5 years and 59 years, respectively. A larger proportion of cases had grade-3 tumors, tumors above 2 cm, and ER-positive/PgR-negative disease (locally assessed). Seven of the cases were randomized to receive L. Median GGI in cases was 785 compared with 669 for controls. Overall median follow-up time, based upon Kaplan-Meier estimates, was 27.9 months [range: 11 to 66 months].

**Table 2:** Univariate comparisons of baseline characteristics.

	**Case/Control**					
			
	**Case**		**Control**		**Overall**	
		
**Characteristic**	**N**	**(%)**	**N**	**(%)**	**N**	P-value*
**Menopausal category**						
						
**Postmen. before chemo**	9	90.0	34	89.5	43	
	
**Postmen. after chemo**	1	10.0	4	10.5	5	0.99

**Tumor size**						
						
**≤ 2 cm**	1	10.0	15	39.5	16	
	
**> 2 cm**	9	90.0	23	60.6	32	0.13

**Tumor grade**	.	.				
						
**Grade 1**			2	5.3	2	
	
**Grade 2**	3	30.0	27	71.1	30	
	
**Grade 3**	7	70.0	9	23.7	16	0.03

**Nodal status**						
						
**N-/Nx**	4	40.0	14	36.8	18	
	
**N+**	6	60.0	24	63.2	30	0.99

**ER and PgR status**						
						
**ER-pos/PgR-pos**	5	50.0	33	86.8	38	
	
**ER-pos/PgR-neg**	5	50.0	5	13.2	10	0.02

**HER2 Positive**						
	
**No**	8	80.0	29	76.3	37	
	
**Yes**	1	10.0	1	2.6	2	
	
**Unknown**	1	10.0	8	21.0	9	0.41

**Local therapy**						
						
**BCS and radiotherapy**	5	50. 0	24	63.2	29	
	
**BCS and no radiotherapy**	.	.	1	2.6	1	
	
**Mastectomy and radiotherapy**	4	40.0	9	23.7	13	
	
**Mastectomy and no radiotherapy**	1	10.0	4	10.5	5	0.73

**Adjuvant or neoadjuvant chemo**						
						
**No**	6	60.0	24	63.2	30	
	
**Yes**	4	40.0	14	36.8	18	0.99

**Treatment**						
						
**Letrozole**	7	70.0	21	55.3	28	
	
**Tamoxifen**	3	30.0	17	44.7	20	0.49

*Fisher's exact p-value

**Table 3:** Univariate comparisons of other prognostic factors.

	**Case/Control**									
					
	**Case**			**Control**			**Overall**			
		
	**Median**	**Min**	**Max**	**Median**	**Min**	**Max**	**Median**	**Min**	**Max**	P-value*
**Age at randomization**	62.5	52	70	59	46	78	59.5	46	78	0.81

**GGI**	785.10	584.95	854.21	668.91	550.03	872.28	683.23	550.03	872.28	0.02

*Wilcoxon rank-sum

### Overall effect of the Genomic Grade on the hazard of relapse

To estimate the overall effect of GGI upon hazard of relapse, a Cox model, stratified by the four combinations of treatment and nodal status, was employed with the GGI as a continuous, linear predictor. The GGI was significantly and linearly related to risk of relapse: each 10-unit increase in GGI resulted in an increase of approximately 11% in the hazard rate (95% CI 3% to 21%, log-rank p-value = 0.02).

Since there is no defined cutoff to classify the samples into high and low GGI categories, we considered the median value (683.2) as the cut point. The hazard of a relapse for patients with high GGI was not statistically different from the hazard with low GGI (hazard ratio: 4.55, 95% CI 0.95 to 21.7, log-rank p-value = 0.06), although the probability is small enough to provide evidence that there is a relationship between GGI classification (when divided at the median GGI value) and relapse.

### Effect of the Genomic Grade on the hazard of relapse according to follow-up time

**Figure 1:**
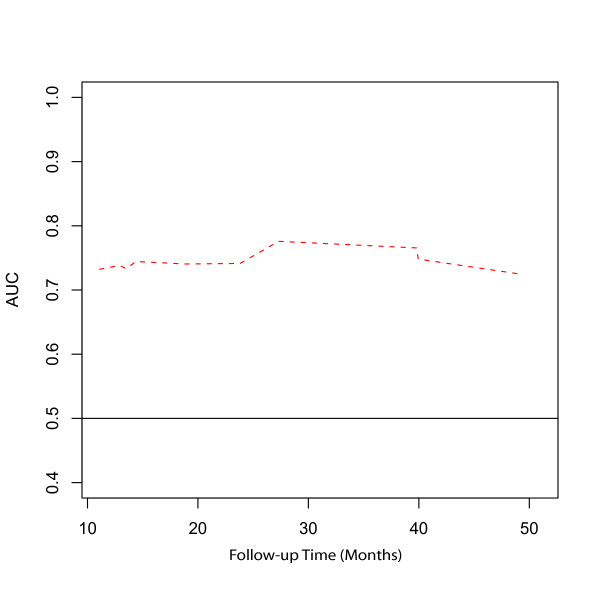
**The predictive accuracy of the GGI estimated using time-dependent area under the curve (AUC) of the ROC curves**.

The prognostic potential of the GGI was assessed using Cox proportional hazards regression with GGI as the single covariate to estimate time-specific ROC curves. ROC curves were constructed for times between 0 and 50 months and the areas under the ROC curves were then plotted to obtain the AUC(t) function (100% = perfect classification, 50% = no discrimination). Estimates of AUC(t) are shown in Figure 1. Over the first 24 months of follow-up, the AUC(t) ranged between 73% and 74%. This may be interpreted to say that for any time, *t*, between 0 and 24 months, the probability was at least 73% that a patient who relapsed at time *t *had a GGI score greater than a patient who had not relapsed at time *t*. AUC(t) reached a maximum value of 77.6% at 27 months, with maximal discrimination occurring at approximately the median follow-up time observed in the data.

**Figure 2:**
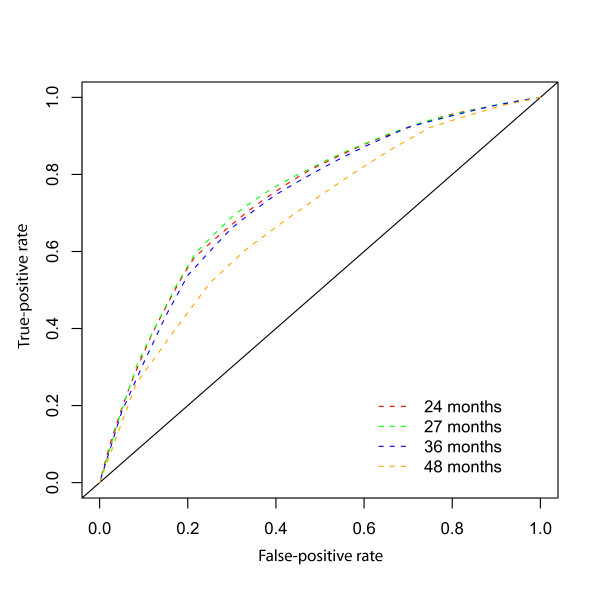
**ROC Curves at 24, 27, 36, and 48 months for GGI Data**.

Figure 2 shows ROC curves at 24, 27, 36, and 48 months based on GGI. The ROC curves also show that predictive accuracy peaks at approximately 27 months and then decreases with increasing time from baseline. The ROC curves may be used to compare the sensitivities resulting from a fixed false-positive rate. For example, controlling the false-positive rate at 20% leads to a sensitivity of 55.5% at 24 months, 56% at 27 months, and 54% and 43% at 36 and 48 months, respectively. Table 4 summarizes true- and false-positive rates for the GGI divided at the median at 24, 27, 36 and 48 months after enrollment. The accuracy summaries (AUC) suggest good discriminatory potential of the GGI within 36 months of enrollment, with the best discrimination occurring near the median follow time.

**Table 4:** Summary of Estimated True- and False-positive Rates over Time.

**GGI value (%-ile)**	**24 Months**		**27 Months**		**36 Months**		**48 Months**	
	
	TP rate	FP rate	TP rate	FP rate	TP rate	FP rate	TP rate	FP rate
**median**	0.82	0.50	0.79	0.41	0.78	0.45	0.68	0.42

**AUC**	0.74		0.776		0.73		0.68	

TP = True-positive; FP = False-positive

### Effect of the Genomic Grade on the hazard of relapse according to treatment

To estimate the effect of GGI upon relapse for each treatment assignment, exploratory subgroup comparisons of times to relapse according to GGI were made separately for letrozole and tamoxifen. For patients randomized to receive L, the hazard of relapse in the patients with GGI above the median was 4.8 times the hazard of patients with GGI below (95% CI: 1.01 to 22.9, p = 0.048). With only 3 cases in the sample receiving T, there was no evidence of a difference in the risk of relapse in that treatment group between high and low GGI.

### Effect of the Genomic Grade on the hazard of relapse according to the nodal status

To estimate the effect of GGI upon relapse for each nodal status, comparisons of times to relapse according to GGI were made separately for subgroups defined by N+ and Nx/N-. For patients with node-positive disease, the hazard of relapse in the patients with GGI above the median was 5.5 times the hazard of patients with GGI below (95% CI: 1.12 to 26.9, p = 0.04). There was no evidence of a difference in the risk of relapse between high and low GGI for patients with node-negative disease, which might be explained by the small number of node-negative cases.

## Discussion

The efficacy of aromatase inhibitors in treating hormone-dependent breast cancer patients has been demonstrated in several clinical trials, where a significant increase in disease-free survival has been shown using third-generation aromatase inhibitors [4-8,13]. However, as with tamoxifen, resistance to these therapies does develop and patients recur. We recently showed that tamoxifen-treated patients whose tumors were of high genomic grade were associated with a worse outcome than those with a low GGI. However, since the actions of tamoxifen (ER antagonist) and aromatase inhibitors (prevention of estrogen synthesis) are inherently different, we sought in this study to assess whether high GGI levels would also be associated with worse outcome in patients treated with aromatase inhibitors.

Altogether, these results confirm our previous findings, i.e. that the GGI is a good predictor of relapse in ER-positive breast cancer patients [3]. Indeed, this analysis supports the GGI as a good predictor of relapse in the sample of 48 postmenopausal patients with hormone-positive breast cancer of the prospective BIG 1–98 trial with available frozen tissue and suggests that higher values of the GGI are associated with an increase in the hazard of a relapse, with each 10-unit increase in GGI resulting in an approximate 11% increase in the hazard rate. Also considering the GGI as a binary variable defined by the median value, provided evidence for a relationship between high GGI values and increase risk of relapse. Using time-dependent, incident/dynamic, ROC/AUC methodology, estimated AUC values between 73% and 78%, occurring during the first 36 months of enrollment, suggest that the GGI has good predictive ability for relapse, with the best predictive ability occurring at approximately 27 months. However, false-positive rates using the GGI were high, remaining above 40%.

Exploratory subgroup analyses within nodal status or treatment suggest that high values of GGI indicate worse outcomes. When the performance of GGI was compared within nodal status, the hazard of relapse was significantly greater for patients with GGI at or above the median within the subgroup of patients with node-positive disease.

Analyses of GGI performance within treatment assignment were somewhat limited, due to the small number of cases with available samples for patients treated with tamoxifen. Within the subgroup of patients randomized to treatment with letrozole, the hazard of relapse was significantly greater for patients with GGI at or above the median when compared to patients with GGI values below.

## Conclusion

These results, together with our previous retrospective data on tamoxifen-treated patients, suggest that the ER-positive high-genomic grade patients would require another treatment strategy.

Although very promising, our results are exploratory and need to be confirmed. This is planned on a larger number of samples from this same BIG 1–98 trial using the simplified GGI, which is represented by only four genes and can be tested by RT-PCR on formalin-fixed paraffin-embedded tissues (FFPE) that, in contrast to frozen samples, are routinely available in all hospitals. We will then also be able to investigate whether, as reported recently by Viale et al. on 2685 women from this same trial [14], highly proliferative tumors, captured in their study by Ki-67 protein expression, show the greatest differential benefit of L over T. Also, since researchers recently demonstrated through in-vitro studies that resistance to aromatase inhibitors is an extremely complex phenomenon, which does vary between anastrazole, letrozole and exemestane [15,16], we should be cautious before generalizing these results to all third-generation aromatase inhibitors.

## Competing interests

C. Sotiriou and M. Piccart are named inventors on a patent application for the Gene expression Grade Index used in this study. There are no other conflicts of interest.

## Authors' contributions

CD and CS designed the overall study. FL performed the microarray experiments. BHK processed the microarray data. AG-H and RG performed the statistical analysis. MP, PN, RP, MC, AS and CS provided expertise in clinical breast oncology. CD wrote the manuscript. All authors approved the final version of the manuscript. All authors contributed to the preparation of the manuscript. 

## Pre-publication history

The pre-publication history for this paper can be accessed here:


